# Sexual Perversion—I

**Published:** 1909-01-16

**Authors:** 


### Mental Diseases.

### SEXUAL PERVERSION—I.

Amongst the sane of both sexes, sexual perversion is, unfortunately, more frequently found to exist than is generally supposed, and is common, as would be expected, in our asylum population. The perversion assumes many forms, some varieties being mild and comparatively inoffensive, others revolting or regarded as criminal, but all unhealthy from the point of view of mental hygiene. At the present time sexual subjects are being brought prominently before the public in "novels," supposed by some to be smart and by others to be of value in elucidating social problems, but which can only be truly described as "dirt," and which, rather than indicating an advance in scientific thought, merely prove the need of a literary censor. Many books, too, have been published within recent years dealing with the different forms of sexual perversion, some written in a scientific spirit, and others more from a popular standpoint. Of these works those of the latter variety can only be condemned, whilst those of the former kind, though really useful to a few medical specialists, seem more to occupy a place as encyclopædic works for the legal profession and for the police. It must not be forgotten that for a discussion of any value on these subjects a good knowledge of physiology, as well as of psychology is necessary, and that this knowledge is not possessed by the general public. Further, that, as a rule, those who do possess this knowledge will not write on these matters for the public. It may be useful, therefore, to take a short review, not of a mere epitome of the different varieties of sexual perversion, with the name of each and examples of it, but of the subject broadly, very briefly directing attention to its cause, prevention, and treatment.

It seems possible at the outset to divide cases of sexual perversion into two main classes—(*a*) those in which it appears to be congenital, and (*b*) those in which it is acquired. Individuals of the former class seem either never to have had any normal sexual instinct or else to have lost it early and easily acquired an unnatural one. The opinion of most physicians who have made this subject their special study seems to be that by far the greater number of cases fall into this class. In these cases there is affective mental abnormality which may be so slight that the individual can control his feelings and never give way to vicious practices, or may be grave, causing him to commit criminal offences. This mental obliquity may again be more widespread, involving the intellectual centres, the patient even being certifiably imbecile. Pathological heredity will probably be found in most of the cases of this class, and the greater number of them will be a danger to the community as sources of contamination to the weak. In this category are sometimes seen people of genius, exceptionally brilliant in certain mental spheres, but unstable, the excessive development of some particular mental traits having taken place at the expense of others. In the second class of cases fall those whose instincts were normal, but who have been contaminated, and have contracted a habit which, as is natural with habits, has become fixed with practice. Amongst the causes in this class are anatomical abnormalities and pathological changes which are sources of irritation, bad habits of life such as errors of diet with sedentary or idle existence, bad companions, literature, etc. Cases are found also in which the perversion is only a symptom of an attack of insanity. The chances of reformation will naturally be better in these cases than in those of the former class, and will be more promising the earlier the case is discovered.

To collect data for an attempt to assign a cause for the trouble, the family history should be closely investigated, with a view to finding any pathological habit, epilepsy, eccentricity, or mental instability in immediate ancestors or their offspring. The confidence of the patient should, if possible, be obtained, and his own account of his instincts, feelings, habits, and sexual history noted. It must be remembered that these patients, like others with mental obliquity, or like those addicted to other bad habits, are not models of veracity, and that often the normal gift of imagination is with them so much hypertrophied that romance may be introduced into the tale "to give an air of verisimilitude to an otherwise bald and uninteresting narrative." The history thus obtained should therefore be supplemented and, if possible, verified by inquiries from others. The mental condition must be examined to see if there is any sign of intellectual or moral deficiency or of recently acquired insanity of which the perversion may be but a symptom. Signs of morbid effeminacy in men and of the opposite in women are interesting, and may corroborate other evidence. Enquiries must be made to ascertain if there has been contamination by bad companions or literature, and concerning ordinary habits of life, diet, work, exercise, amusements, and hobbies. A physical examination must not be omitted to discover any anatomical or pathological abnormality.

